# The Potential of Cell-Penetrating Peptides for mRNA Delivery to Cancer Cells

**DOI:** 10.3390/pharmaceutics14061271

**Published:** 2022-06-15

**Authors:** Yelee Kim, Hyosuk Kim, Eun Hye Kim, Hochung Jang, Yeongji Jang, Sung-Gil Chi, Yoosoo Yang, Sun Hwa Kim

**Affiliations:** 1 Medical Materials Research Center, Biomedical Research Institute, Korea Institute of Science and Technology (KIST), Seoul 02792, Korea; whyj8007@kist.re.kr (Y.K.); hyoseog7@kist.re.kr (H.K.); ehkelly@kist.re.kr (E.H.K.); hjang@kist.re.kr (H.J.); yeongji6073@kist.re.kr (Y.J.); 2 Department of Life Sciences, Korea University, Seoul 02841, Korea; chi6302@korea.ac.kr; 3 Division of Bio-Medical Science and Technology, KIST School, Korea University of Science and Technology, Seoul 02792, Korea

**Keywords:** cell-penetrating peptide, mRNA, cancer therapy, nucleic acid delivery system

## Abstract

In vitro transcribed mRNA for the synthesis of any given protein has shown great potential in cancer gene therapy, especially in cancer vaccines for immunotherapy. To overcome physiological barriers, such as rapid degradation by enzymatic attack and poor cellular uptake due to their large size and hydrophilic properties, many delivery carriers for mRNAs are being investigated for improving the bioavailability of mRNA. Recently, cell-penetrating peptides (CPPs) have received attention as promising tools for gene delivery. In terms of their biocompatibility and the ability to target specific cells with the versatility of peptide sequences, they may provide clues to address the challenges of conventional delivery systems for cancer mRNA delivery. In this study, optimal conditions for the CPP/mRNA complexes were identified in terms of complexation capacity and N/P ratio, and protection against RNase was confirmed. When cancer cells were treated at a concentration of 6.8 nM, which could deliver the highest amount of mRNA without toxicity, the amphipathic CPP/mRNA complexes with a size less than 200 nm showed high cellular uptake and protein expression. With advances in our understanding of CPPs, CPPs designed to target tumor tissues will be promising for use in developing a new class of mRNA delivery vehicles in cancer therapy.

## 1. Introduction

In cancer therapy, protein expression has been used as a direct approach in the reconstitution of defective genes [1]. Nucleic acid-based therapies, i.e., plasmid DNA and messenger RNA (mRNA) therapies, pave the way for efficacious and safe biologics [2,3]. Nucleic acid-based therapies have superior safety, efficacy and specificity over existing therapeutics, such as small molecules and antibodies [4]. Of particular note is that mRNA-based gene therapies have benefits when compared with therapeutics based on other types of nucleic acids [5]. While DNA-based gene therapy has obstacles of random insertion into the host genome that can induce carcinogenesis, mRNA-based approaches only target cytoplasmic delivery, circumventing the risk of genomic integration [6]. Further, mRNA could be prepared in a cell-free system by in vitro transcription (IVT), avoiding the quality and safety issues associated with production using microorganisms or cultured cells [7]. This allows for simple downstream purification and rapid and cost-effective manufacturing.

mRNA-based gene therapy shows promising potential in novel vaccine development against infectious diseases and new therapeutics for a wide range of indications and diseases, including cancer [8]. Recently, mRNA-based cancer vaccines which express tumor-associated antigen (TAA) have gained tremendous attention as alternative cancer immunotherapy because they can specifically attack and destroy cancer cells through immune stimulation [9]. Moreover, exogenous mRNA can elicit interferon-related pathways and function as self-adjuvants. For example, CureVac has developed a TAA mRNA vaccine in which TAA mRNA is co-delivered with protamine/mRNA complexes which have an adjuvant-like effect [10]. However, the effective delivery of mRNA to the cytoplasm continues to be the main obstacle, particularly for the systemic administration of mRNA [11]. The high negative charge and large molecular weight (10^5^–10^6^ Da) of mRNA limits the permeation across cell membranes [12]. In addition, mRNA has a short half-life of approximately 7 h, naked mRNA is highly prone to degradation by exonucleases and endonucleases [13]. Therefore, the application of mRNA in cancer therapy critically depends on efficient delivery systems [14]. Typically, for cytosolic delivery, IVT mRNA is complexed and formulated with a delivery vehicle that can protect the mRNA from rapid degradation and promote cellular uptake [15]. In this regard, lipid- and polymer-based systems have been extensively studied, especially through the development of COVID-19 vaccines [16,17].

Presently, lipid-based nanoparticles (lipid nanoparticles, LNPs) are mostly used for delivering mRNA [18]. Among the components of LNPs, ionizable cationic lipids spontaneously encapsulate negatively charged mRNA by a combination of electrostatic and hydrophobic interactions [13,19]. Although LNPs provide a great opportunity for efficient intracellular nucleic acid delivery [18], challenges remain concerning the stability of current mRNA-LNP systems, which must be stored at ultra-low temperatures [20], in addition to LNP-related toxicity and immunogenicity [21]. As the field of LNP-technology has reached saturation, and ‘Arbutus’ and ‘Genevant’ currently monopolize the mRNA-LNP market [22], the development of new mRNA delivery platform technology is seriously required.

New mRNA delivery systems should meet simple production and improved biocompatibility. In terms of high transfection efficiency, safety and low production cost, peptide-based delivery offers great promise [23]. Cell-penetrating peptides (CPPs) are usually 4–40 amino acids long and possess cationic or amphipathic motifs that have the ability to cross the cell membrane [24]. They have been used to deliver insoluble small molecule drugs, proteins and nucleic acids to target tissues and cells, and have emerged as non-viral alternatives to viral vectors [25]. Peptide-based mRNA delivery systems are gaining momentum due to the versatility of peptide sequences [8]. Arginine-rich cationic CPPs are known to promote cellular internalization through hydrogen bonding and electrostatic interactions with the cell membrane surface via the guanidinium group of arginine [26,27]. Amphipathic CPPs are also composed of cationic lysine and/or arginine residues with an even distribution of hydrophobic amino acids, such as tryptophan, leucine, alanine and valine [28]. These peptides exhibit high transfection efficiency due to the strong hydrophobic interaction and the penetration into lipid bilayers in the cell membrane [29]. Since most cancer cell surfaces are overall negatively charged, positively charged molecules outperform negatively charged molecules in cellular uptake efficiency [30]. For example, PepFect14, a commercially available CPP has been developed as a platform for ovarian cancer therapy by forming complexes with reporter mRNA [31].

Although more than 1800 kinds of CPPs have recently been discovered and synthesized [32], little is known about their delivery efficiency via direct electrostatic interaction with mRNA. In this paper, we conducted the first comparative study based on the physicochemical properties of CPPs (three cationic CPPs and five amphipathic CPPs, Table 1) capable of forming stable complexes by non-covalent interactions with mRNA. The mRNA-containing nanoparticles were produced by simple electrostatic complexation between mRNA and CPPs containing cationic and amphipathic sequences. Among them, three of the CPP/mRNA complexes were found to induce effective cellular uptake of mRNA and subsequent expression of the reporter protein in CT26.CL25 cancer cells with no apparent toxicity. Collectively, it was found that the presence of hydrophobic moieties, amino acid composition and structure of CPP play an important role in complex formation with mRNA and the transfection efficiency of cancer cells.

## 2. Materials and Methods

### 2.1. Materials

Most experiments were performed using the following two fluorescent reporter genes (both 996 nt in length) commonly used in in vitro fluorescence imaging and flow cytometry. The 5-methoxyuridine-modified mCherry mRNA (L-7203), enhanced green fluorescence protein (EGFP) mRNA (L-7201) and Ovalbumin (OVA) mRNA (L-7610) were purchased from Trilink Biothechnologies (San Diego, CA, USA). Two fluorescent reporter genes (both 996 nt in length) were chosen because they are commonly used for in vitro fluorescence imaging and flow cytometers. TAT peptide (RP20256) was obtained from GenScript (Nanjing, China). Penetratin (AS-61032) was purchased from AnaSpec (Fremont, CA, USA). Pep-1 (ab142343) was purchased from Abcam (Cambridge, MA, USA). Poly-Arginine8 (Arg8), low molecular weight protamine (LMWP), Stearyl-poly-Arginine8 (STR-Arg8), p5RHH and RALA peptides were synthesized by Peptron (Daejeon, Korea). The purity of all peptides was >99%.

### 2.2. CPP/mRNA Complex Formation and Characterization

The mRNA was diluted in RNase-free water at 100 ng/μL and incubated with CPPs (1 μg/μL in RNase-free water) at room temperature (RT) for 1 h at 200 rpm. For in vitro studies, CPP/mRNA complexes were diluted with 1× PBS by adding 10× PBS after complexation. To visualize the complexation of mRNA and CPP, 350 ng of mCherry mRNA was mixed with increasing amounts of CPP and a gel shift assay was performed using a 1% agarose gel. Complexation capacity was measured based on mRNA band intensities by Image J.

Complexation capacity (%) = 100 − ((Band intensity of mRNA)/(Band intensity of initial mRNA) × 100))

A Decomplexation assay was performed using heparin sodium salt (H3149) (Sigma Aldrich, St. Louis, MI, USA). After 1 h complexation of CPP and mRNA, 1–10 μg of heparin was treated at RT for 1 h. The protective ability of CPP/mRNA complexes from RNase was analyzed by treatment with RNase A (50 μg) for 30 min at RT and then loaded onto a 1% agarose gel.

The size and zeta-potential of CPP/mRNA complexes were measured by dynamic light scattering (DLS) using Zetasizer Nano ZS (Malvern Instruments, Malvern, UK); 50 μg of CPPs mixed with an optimized amount of mRNA were diluted in 1 mL of distilled water and then transferred to a cuvette. The morphology and structure of CPP/mRNA complexes were measured by transmission electron microscopy (JEM-1010, Nippon Denshi) at 80 kV. For negative staining, CPP/mRNA complexes were loaded onto a copper mesh grid (TEM-FCF300CU, Sigma) and stained with 1% phosphotungstic acid (P4006, Sigma).

### 2.3. Cell Cultures

HEK293T (human embryonic kidney) and CT26.CL25 (mouse colorectal carcinoma) cell lines were obtained from the American Type Culture Collection (ATCC, Manassas, VA, USA). HEK293T and CT26.CL25 cells were incubated in DMEM (Hyclone, Logan, UT, USA) and RPMI-1640 (Welgene, Gyeongsan, Korea), respectively, both were supplemented with 10% FBS (Atlas Biologicals, Fort Collins, TX, USA) and 1% antibiotic-antimycotic (Gibco, Waltham, MA, USA). All cell lines were incubated at 37 °C with a 5% CO_2_ atmospheric condition.

### 2.4. In Vitro Cytotoxicity of CPP/mRNA Complexes

To evaluate cell viability, CT26.CL25 cells seeded in 96 well plates were treated with different doses of CPP/mRNA complexes for 3 h and the media was changed. After 24 h of treatment, a solution of Cell Counting Kit-8 (Dojindo, Tabaru, Japan) was added and quantified at 450 nm with a SpectraMax 34 microplate reader (Molecular Devices, Sunnyvale, CA, USA).

### 2.5. Cellular Uptake of CPP/mRNA Complexes

For the cellular uptake test, mCherry mRNA was labeled with fluorescein dye using the Label IT nucleic acid labeling kit (MIR 3200) (Mirus Bio, Madison, WI, USA). CT26.CL25 cells plated on a 35 mm glass bottom dish were incubated in 1.5 mL of serum-free media for 2 h. Following the incubation, 3 μg (6.8 nM) of mRNA with Lipofectamine 3000 (ThermoFisher, Waltham, MA, USA) or CPPs were treated for 3 h. After treatment, CT26.CL25 cells were collected with PBS and FITC-positive cells were measured by a CytoFLEX flow cytometer (Beckman Coulter, Palo Alto, CA, USA) using FlowJo v10 software.

### 2.6. Evaluation of CPP/mRNA-Mediated Protein Expression

To measure expression efficiency, cells seeded on a 35 mm glass bottom dish were incubated in 1.5 mL of serum-free media for 2 h. After incubation, mCherry mRNA with Lipofectamine 3000 or CPPs were treated for 3 h, washed, and incubated in complete medium for an additional 21 h. The expression of mCherry protein was visualized with a fluorescence microscope (EVOS M7000, ThermoFisher). The expression efficiency was calculated by counting the number of cells expressing fluorescence in the total number of cells. For quantification of expression efficiency, flow cytometry analyses were examined after 24 h treatment with CPP/EGFP mRNA. CT26.CL25 cells were collected with PBS and EGFP-expressing cells were measured by a CytoFLEX flow cytometer (Beckman Coulter) using FlowJo v10 software.

For western blot analysis, CT26.CL25 cells treated with Lipofectamine or CPP/mRNA for 24 h were lysed with 2X SDS. Subsequently, cell lysates were separated on SDS-polyacrylamide gel and then transferred to a nitrocellulose membrane. The membranes were blocked with 3% skim milk in TBS-T for 1 h and incubated with primary antibodies (OVA and GAPDH) overnight at 4 °C. The membranes were then washed with TBS-T and exposed to secondary antibodies for 1 h at RT. After washing with TBS-T, the membranes were developed using an ECL substrate (Bio-Rad, Hercules, CA, USA) and visualized by iBright 750. The following western blot antibodies were used: OVA (Bio-Rad, #0220-1682, 1:1000), GAPDH (R&D, MAB5718, 1:1000), Anti-Rabbit IgG-HRP antibody (Gene Tex, GTX213110-01, 1:2000) and Anti-mouse IgG-HRP antibody (GebeTex, GTX213111-01, 1:2000).

### 2.7. Statistical Analysis

The statistical analysis was performed using Prism 8.0 (Graphpad, La Jolla, CA, USA). Statistical significance was determined by ANOVA or *t*-test. Statistical significance was considered at a *p*-value less than 0.05.

## 3. Results

### 3.1. Optimization of CPP/mRNA Complex Formation

**Figure 1:**
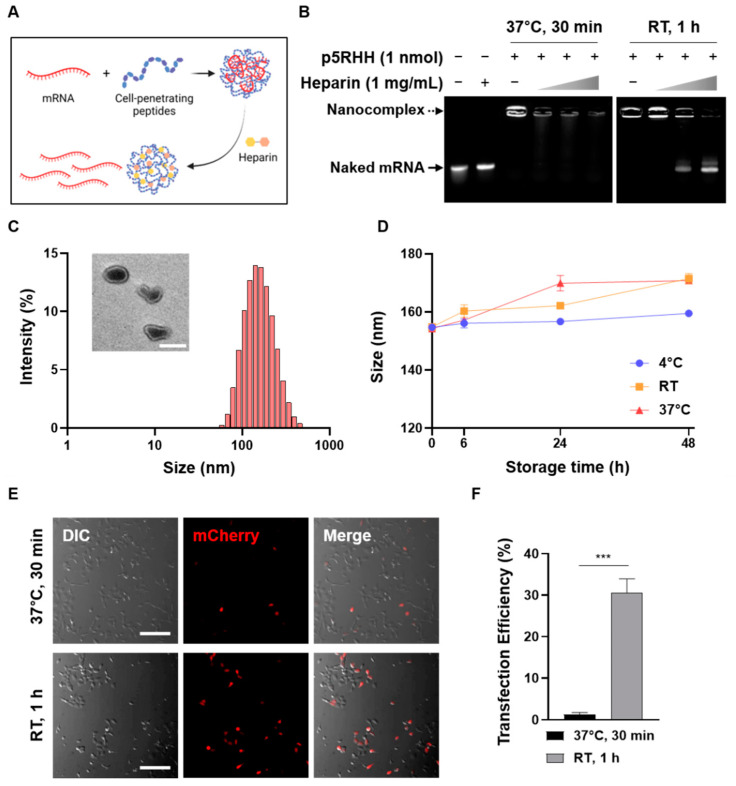
Optimization of CPP/mRNA complex formation. (**A**) Schematic showing CPP/mRNA complexation and heparin-mediated decomplexation. (**B**) Agarose gel images showing the decomplexation of p5RHH/mCherry mRNA complexes incubated under two different conditions. Solid arrow represents naked mCherry mRNA and dashed arrow represents p5RHH/mCherry mRNA complexes. (**C**) Size distribution diagram and representative TEM image of p5RHH/mRNA complexes. Scale bar: 100 nm. (**D**) Size stability of p5RHH/mRNA complexes at various storage temperatures. (**E**) Representative fluorescence images of HEK293T cells after treatment with 3 μg of p5RHH/mCherry mRNA complexes mixed with the indicated conditions. Scale bar: 200 μm. (**F**) Graph showing percentage of mCherry-positive cells. Data are presented as the mean ± SD (*n* = 3). (*** *p* < 0.001; Two-sided *t*-test).

To optimize the conditions for the electrostatic formation of the CPP/mRNA complexes, mCherry mRNA and CPP (p5RHH) mixed under various conditions were treated with highly negatively charged heparin (Figure 1A). First, we confirmed that the complexation between p5RHH and mRNA was not observed under the conditions of 30 min at RT (data not shown), but the p5RHH/mRNA complexes could be successfully formed after an incubation of 30 min at 37 °C or 1 h at RT (Figure 1B). However, when p5RHH/mRNA was decomplexed by heparin, the mRNA of the complexes incubated at 37 °C for 30 min was dissociated and damaged, whereas the mRNA of the complexes reacted at RT for 1 h was intact (Figure 1B). The size of complexes was then analyzed by dynamic light scattering (DLS), and their morphologies were observed by transmission electron microscopy (TEM) (Figure 1C). Most p5RHH/mRNA complexes showed a size distribution of about 100~200 nm with a non-uniform shape. The dispersion stability of the nanoparticles is one of the prerequisites for effective in vivo delivery and commercial applications. Thus, the long-term stability of nanocomplexes at different temperatures was monitored and revealed that there was no significant change in complex size even after 48 h under all temperature conditions (Figure 1D).

Next, we compared the transfection efficiency of two different p5RHH/mRNA complexes in HEK293T cells by fluorescence microscopy. As shown in Figure 1D, it was confirmed that the complexes reacted for 1 h at RT showed more mCherry-positive cells. Therefore, in all subsequent experiments, a reaction condition for 1 h at RT was used to form the CPP/mRNA complexes.

### 3.2. Screening of CPPs Suitable for Complexation with mRNA

In this work, we compared the ability of three types of cationic CPPs (Arg8, TAT and LMWP) and five types of amphipathic CPPs (STR-Arg8, p5RHH, RALA, Pep-1 and Penetratin) as mRNA delivery carriers (Table 1). The cationic CPPs (Arg8, TAT and LMWP) have been extensively studied in the field of gene delivery because they can form nano-sized particles with nucleic acids through their cationic properties [33,34,35,36,37,38]. The amphipathic CPPs (p5RHH, RALA, Pep-1 and Penetratin) have also been found to be efficient delivery vehicles for DNA and/or RNA because they can interact with nucleic acids as cationic moieties [23,39,40] and exhibit strong hydrophobic interactions with cell membranes [41]. Additionally, in order to confirm the effect of hydrophobicity in CPP, STR-Arg8 in which a hydrophobic stearyl moiety was introduced into a cationic Arg8 was also investigated.

**Figure 2:**
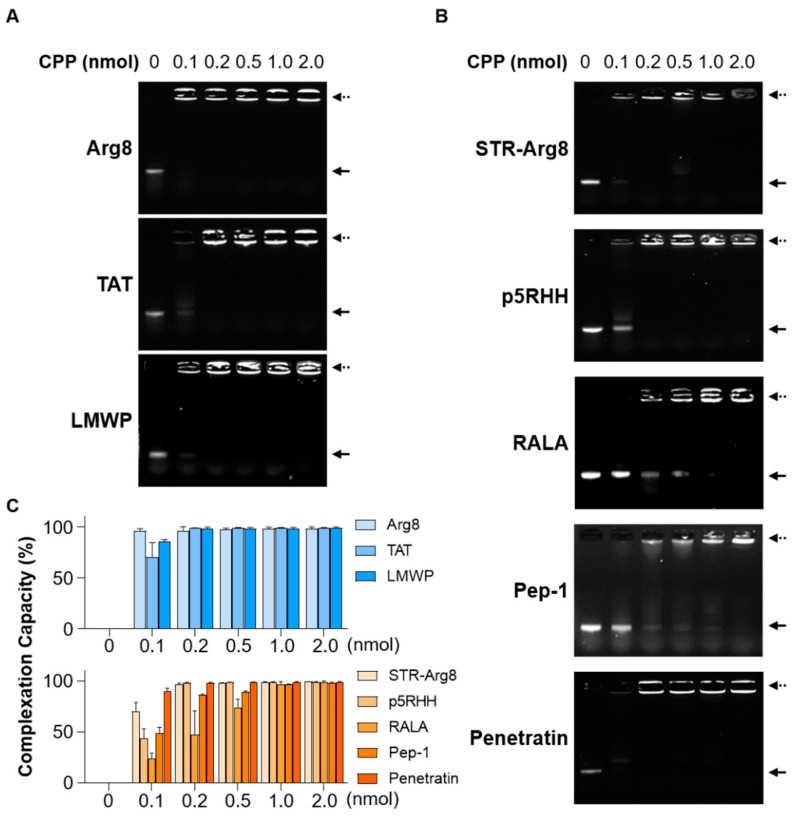
Formation of complexes between mRNA and CPPs. Representative agarose gel images showing mCherry mRNA (350 ng) complexed with the increasing amounts of cationic CPPs (**A**) and amphipathic CPPs (**B**). Solid arrow represents naked mCherry mRNA and dashed arrow represents CPP/mCherry mRNA complexes. (**C**) Complexation capacity of cationic (upper graph) and amphipathic (lower graph) CPPs based on mRNA band intensities. Data are presented as the mean ± SD (*n* = 3).

The complexation of cationic and amphipathic CPP/mRNA was validated by mixing CPPs (0–2.0 nmol) with mRNA (350 ng) using a gel shift assay (Figure 2A,B). In all CPPs, as the amount of peptide increased, the amount of mRNA incorporated into the CPP/mRNA complexes increased. Next, to compare the complexation capacity of CPPs, complexations with a broad range of CPP to mRNA ratios were calculated based on the mRNA band intensities (Figure 2C). Most CPPs showed a complexation capacity of 99% or more at 0.2 nmol, whereas Pep-1 and RALA formed complete complexes at 1.0 nmol. Notably, Pep-1 and RALA exhibited the lowest ratios of net charge to peptide length (14.2 and 16.6%, respectively, Table 1). These results support that the content of cationic amino acids with respect to the total length of the peptide might be important for the complexation efficiency of CPPs [42].

CPPs can deliver cargoes up to 200 nm in diameter [43]. Thus, the capacity of CPPs to condense mRNA into nano-sized particles (<200 nm) was verified; the ratio of the number of positively charged amine groups in each peptide to the number of anionic phosphate groups in the mRNA (N/P ratio) was calculated, and the particle size and zeta-potential were measured by Zetasizer Nano ZS (Table 2). The lowest N/P ratios of cationic CPPs to form nanocomplexes of around 150 nm were 1.5 for Arg8, 3.9 for TAT and 1.9 for LMWP. For amphipathic CPPs (STR-Arg8, p5RHH and RALA), particle sizes below 200 nm were formed at higher N/P ratios than those of cationic CPPs. In the case of Pep-1 and Penetratin, irregular large particles were formed even though N/P ratios were increased to more than 10. Recently, the conformational state of amphipathic CPPs, which is determined by peptide sequences and arrangement, has been shown to play an important role in peptide-oligonucleotide interactions [44,45]. For example, among the various CPPs designed based on different tryptophan distributions, only those displaying unstructured random coils formed irregular large particles with siRNA, unlike the rest displaying α-helical structures [45]. Since Pep-1 and Penetratin mainly have a random coiled structure in water and are highly versatile depending on their microenvironment [46,47], these structural properties may hinder the formation of compact nanocomplexes with mRNA. Despite the difference in size, the zeta-potentials of the different CPP/mRNA nanocomplexes showed effective surface charges between +5~30 mV.

**Figure 3:**
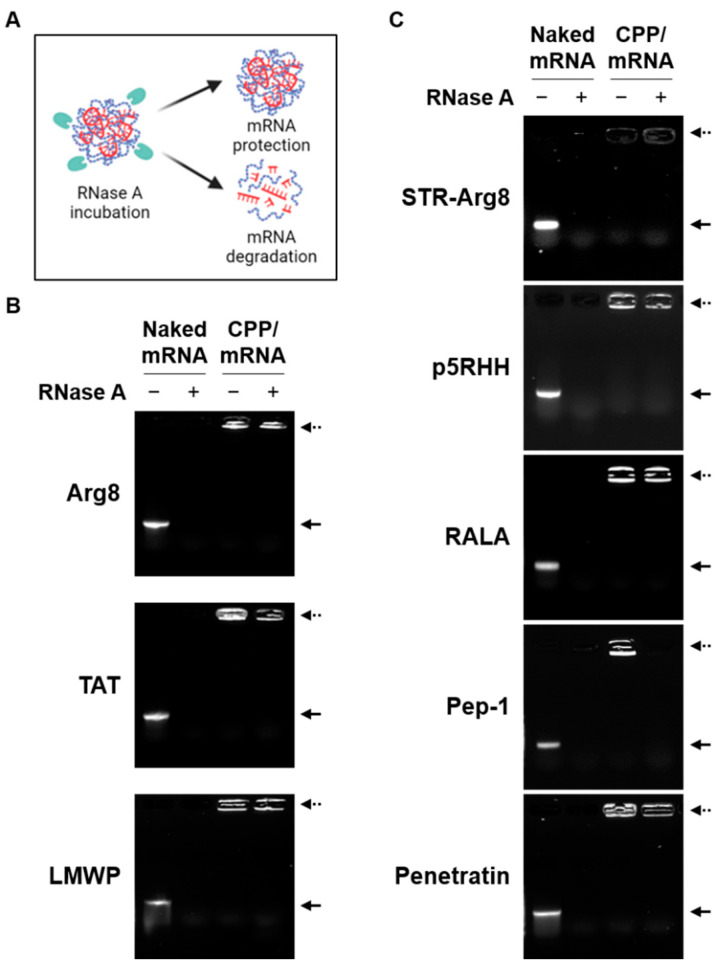
The protective ability of CPP/mRNA complexes. (**A**) Schematic showing the protective ability of the CPP/mRNA complexes from RNase. Representative agarose gel images showing naked mRNA and cationic (**B**) or amphipathic (**C**) CPP/mRNA complexes (with or without RNase A incubation). Solid arrow represents naked mCherry mRNA and dashed arrow represents CPP/mCherry mRNA complexes.

The complex formation with CPPs can protect mRNA from endonuclease-mediated degradation during cellular uptake [25]. As shown in Figure 3, while naked mRNA was completely degraded by RNase, all cationic CPPs and most amphipathic CPPs were found to protect encapsulated mRNA. Notably, only mRNA complexed with Pep-1 was degraded by RNase. Because only Pep-1/mRNA complexes exhibited a particle size greater than 500 nm (Table 2), this suggests that the unstable nano-complexation of mRNA and Pep-1 results in poor protection ability. Collectively, the amino acid composition and structural properties of CPPs can affect their ability to form stable and nano-sized complexes with mRNA.

### 3.3. Intracellular Cytotoxicity of CPP/mRNA Complexes

**Figure 4:**
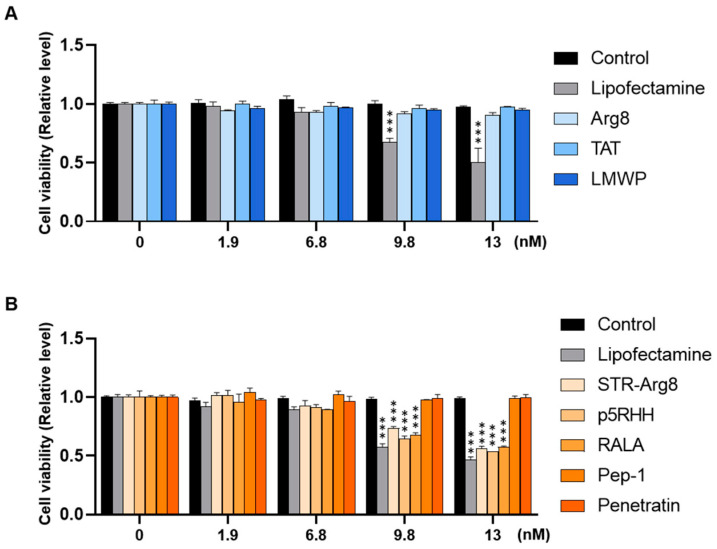
Intracellular cytotoxicity of CPP/mRNA complexes. Viability of CT26.CL25 cells treated with various concentrations of cationic (**A**) or amphipathic (**B**) CPP/mCherry mRNA were measured using the CCK-8 kit, respectively. Data are presented as the mean ± SD (*n* = 3). *p*-values are displayed only in groups below 80% of viability (minimum acceptable viability) compared to Controls. (*** *p* < 0.001; Two-sided *t*-test).

To examine the cytotoxicity of CPP/mRNA complexes, CT26.CL25 cancer cells were treated with CPP/mRNA complexes at various concentrations (0, 1.9, 6.8, 9.8 and 13 nM based on mRNA concentration). With the minimum acceptable viability set at 80% (according to ISO 10993-5), amphipathic CPPs showed significant cytotoxicity from 9.8 nM of mRNA, whereas little toxic impact was observed in cationic CPP-treated groups (Figure 4A,B). The cytotoxicity of lipofectamine 3000, a widely used transfection reagent, was higher than that of CPPs at almost all concentrations. Since cell viability similar to that of Control was confirmed in all nanocomplexes containing 6.8 nM mRNA (Figure 4), subsequent in vitro experiments were then performed at the fixed concentration.

### 3.4. In Vitro Cellular Uptake of CPP/mRNA Complexes

**Figure 5:**
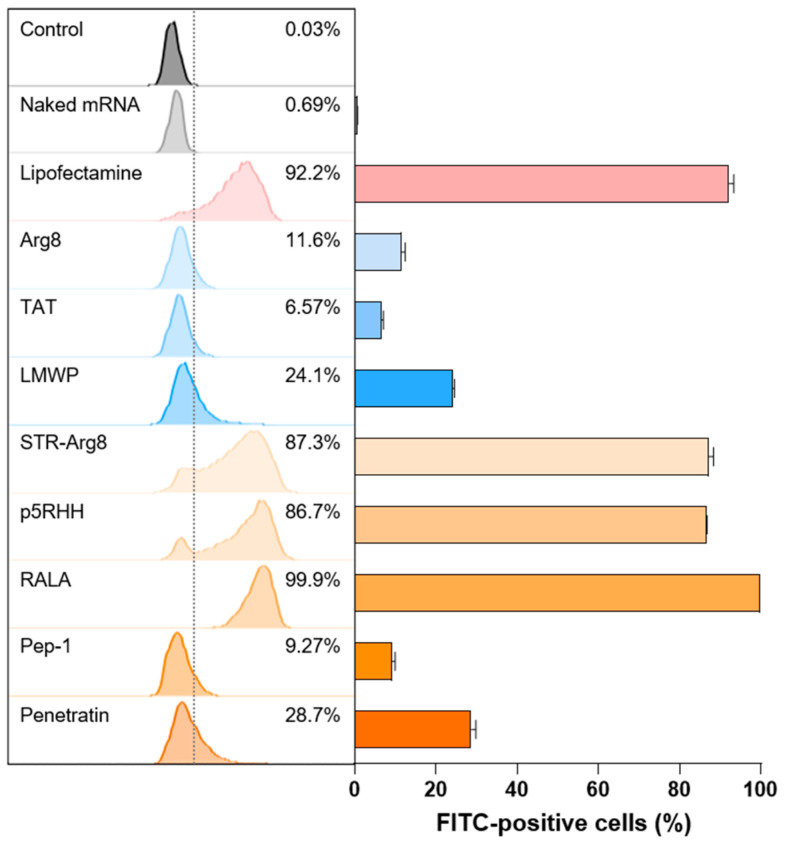
Cellular uptake of CPP/mRNA complexes. Representative flow cytometry histograms and quantified graph of FITC-positive CL26.CL25 cells transfected with CPP/Fluorescein-labeled mCherry mRNA complexes (6.8 nM). Data are presented as the mean ± SD (*n* = 3).

To investigate whether CPP/mRNA complexes improve the delivery of mRNA into the cytosol, we examined in vitro cellular uptake in CT26.CL25 cancer cells. The mCherry mRNA was labeled with fluorescein dye and complexed with CPPs. Then CPP/fluorescein-labeled mCherry mRNA was added to cancer cells for 3 h and observed using flow cytometry (Figure 5). Because the negative charge of mRNA electrostatically repulses the anionic membrane [12], naked mRNA showed no cellular uptake. In addition, all three cationic CPPs exhibited poor uptake efficiency (11.6% for Arg8, 6.57% for TAT and 24.1% for LMWP). These results may correspond to previous studies that electrostatic interactions between CPPs and nucleic acids could mask the cationic motifs of CPP and thus inhibit its internalization function [48]. Additionally, the two amphipathic CPPs, Pep-1 and Penetratin, which form irregular large particles showed a low cellular uptake of 9.72 and 28.7%, respectively.

In contrast, comparable or higher mRNA uptake efficiencies to Lipofectamine were observed for STR-Arg8 (83.7%), p5RHH (86.7%) and RALA (99.9%)-mediated mRNA complexes. In particular, compared with Arg8/mRNA, the uptake rate of STR-Arg8/mRNA complexes increased approximately 7.5-fold, suggesting that hydrophobicity plays a critical role in cellular uptake. Moreover, p5RHH and RALA promote effective membrane permeability because of their facial amphiphilicity, which indicates a segregated structural type with cationic moieties on one side and hydrophobic moieties on the other side [49]. Since the cellular uptake of nanoparticles disrupts membrane stability and leads to cytotoxicity [50], our results confirmed that the cytotoxicity (Figure 4) and uptake efficiencies are strongly correlated. Taken together, these data suggest that amphipathic CPPs capable of forming nano-sized (<200) complexes with mRNA are suitable for cellular uptake.

### 3.5. Evaluation of CPP/mRNA-Mediated Protein Expression

**Figure 6:**
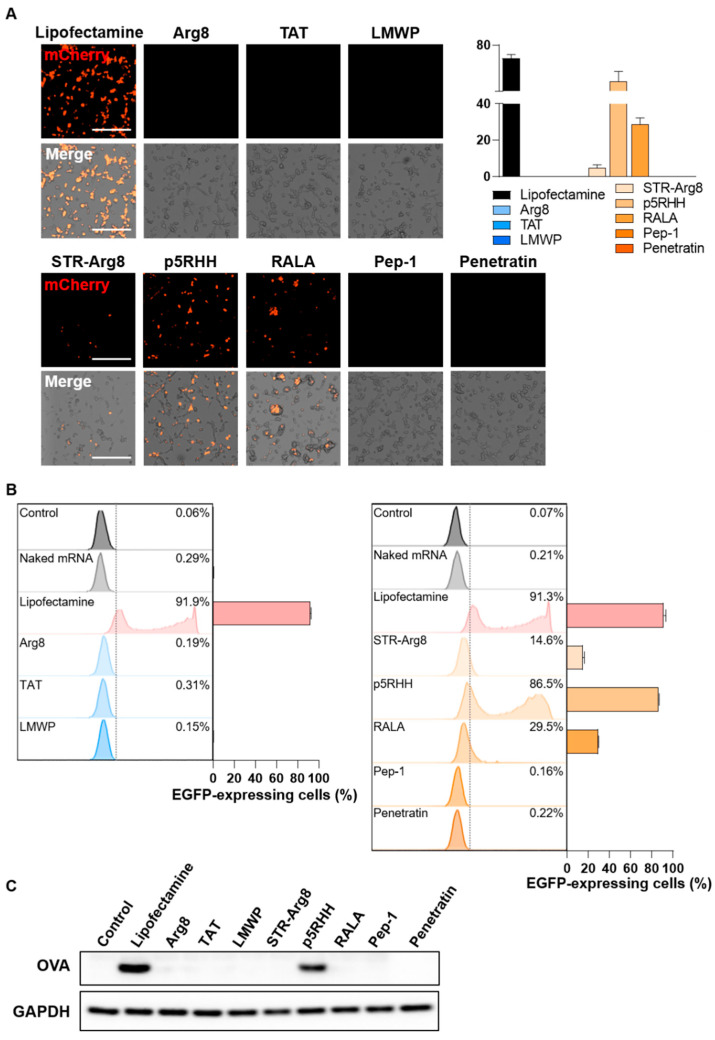
Evaluation of CPP/mRNA-mediated protein expression. (**A**) Representative fluorescence images and a quantitative graph showing mCherry-positive CT26.CL25 cells 24 h after treatment with CPP/mCherry mRNA complexes (6.8 nM). Scale bar: 275 μm. Data are presented as the mean ± SD (*n* = 3). (**B**) Representative flow cytometry histograms and graphs of EGFP-positive CL26.CL25 cells transfected with CPP/EGFP mRNA complexes (6.8 nM). Data are presented as the mean ± SD (*n* = 3). (**C**) Western blot analysis of the expression levels of OVA and GAPDH in CT26.CL25 cells.

Next, we validated the efficiency of CPP/mRNA-mediated protein expression in CT26.CL25 cancer cells; 24 h after CPP/mRNA complexes treatment, the expression of mCherry protein was analyzed by fluorescence microscopy (Figure 6A). In the group transfected with the cationic CPP-derived complexes, which showed poor cellular uptake, no mCherry-expressing cells were observed. Additionally, the amphipathic Pep-1 and Penetratin complexes were shown to be incapable of protein expression because of low cellular uptake. In contrast, 4.9, 56.3 and 28.9% of mCherry-positive cells were observed in the STR-Arg8, p5RHH and RALA complexes treatment groups, respectively.

To more precisely quantify the cells expressing the fluorescent protein, we confirmed the number of EGFP-positive cells by flow cytometry (Figure 6B). Consistent with the fluorescence image results, less than 1% EGFP-positive cells were found in all cationic CPPs and two amphipathic CPPs (Pep-1 and Penetratin) treated groups. On the other hand, the treatment of STR-Arg8, p5RHH and RALA complexes induced EGFP expressions of 14.6, 86.5 and 29.5%, respectively. Notably, the transfection efficiency of p5RHH was as effective as that of Lipofectamine, and this result is consistent with previous reports that p5RHH/siRNA or mRNA nanocomplexes effectively facilitate endosomal lysis and escape of RNAs into the cytoplasm [40,51]. However, STR-Arg8 and RALA showed relatively low expression efficiency despite a high cellular uptake rate of >85%. Failure to escape endosomes or excessively tight electrostatic interactions between CPP and mRNA may be responsible for limiting intracellular mRNA release and translation. Furthermore, we assessed the cellular expression levels of the Ovalbumin (OVA) antigen delivered by the CPP/mRNA complexes by western blot analysis, demonstrating that only p5RHH/mRNA complexes promote OVA expression comparable to the Lipofectamine-treated group (Figure 6C).

## 4. Discussion and Conclusions

To date, the mRNA delivery field has focused on LNP, despite various polymers that have been studied [17]. Of them, CPPs are widely explored as delivery platforms of nucleic acids (e.g., plasmid DNA and siRNA), but few studies about CPP-mediated mRNA delivery were discovered [23]. In this paper, we highlight the distinctive features and potentials of CPP/mRNA non-covalent complexation as delivery systems for cancer cells. Covalent strategies ensure high transfection efficiency through a strong association between CPPs and mRNA. Still, the endosomal escape of mRNA may be difficult, and the covalent-linking procedure is labor-intensive and time-consuming [52]. Compared to the covalent strategy, the non-covalent method, which occurs between the anionic phosphate group of mRNA and the cationic amino acid of CPP, is easier to use, produce, and preserve the function of mRNA [53]. For example, Udhayakumar et al. found that amphipathic CPP RALA electrostatically complexed with antigen-encoding mRNA was successfully delivered to immune cells and facilitated mRNA escape from the endosomes to the cytosol, eliciting cytotoxic T cell responses [39]. In the context of cancer therapy, non-covalently conjugated Pepfect14-mRNA nanoparticles are known to deliver mRNA into different cell types, such as tumor cells, fibroblast and immune cells in the peritoneal ovarian tumor microenvironment [31]. As such, papers on non-covalent mRNA delivery mediated by CPPs have been published, but there is no comparative study on the delivery efficiency for cancer cells according to CPP characteristics.

Here, we compared the ability of cationic CPPs and amphipathic CPPs via non-covalent methods as carriers for mRNA. Various conditions for CPP/mRNA complexation were tested and conditions were optimized for 1h at RT where mRNA stability was maintained (Figure 1A). Then, the size and zeta potential of the complexes suitable for cellular uptake according to the N/P ratio were confirmed. We found that only Pep-1 and Penetratin, which mainly exhibit a random coiled conformation, formed irregular particles larger than 200 nm (Table 2). Because the conformational state of amphipathic CPP has been shown to play a critical role in efficient complex formation with nucleic acids, appropriate peptide sequence design (e.g., the adoption of different positions of tryptophan residues or amino acids that influence the protein folding) and modeling of peptide conformation will allow for the prediction of efficient complexation with mRNA [44,45,47]. Additionally, compact complexation between CPP and mRNA provides protection against endonuclease-mediated degradation (Figure 3).

Multiple studies have identified that the hydrophobic residues of CPP can interact with the plasma membrane and facilitate penetration into cells [54]. We also revealed that the hydrophobic portion of CPPs plays a critical role in increasing the uptake of mRNA into cells (Figure 5). Because their cellular uptake disrupts the stability of the membrane and promotes cytotoxicity [50], we confirmed that the delivery efficiency correlates with cytotoxicity (Figure 4). However, mRNA translation efficiency could vary depending on the type of amphipathic structure (Figure 6). In addition, the failure of endosomal escape or excessively tight electrostatic interactions between CPPs and mRNA could limit the intracellular release or translation of mRNA. To improve endosomal escape efficiencies, tumor-specific targeting and stabilization of mRNA for prolonged protein expression, various functionalities could be introduced into CPPs by tuning the sequence and composition of amino acids.

In conclusion, our results showed that amphipathic CPPs exhibited more effective uptake and transfection capacity than cationic CPPs. Notably, the p5RHH/mRNA complexes could be selected for potential cancer therapy. With the continuous research on the application technology of CPPs, the clinical application of CPPs for cancer therapy is expected in the near future.

## Figures and Tables

**Table 1:** Amino acid sequence, molecular weight and net charge (at pH 7.0) of cationic and amphipathic CPPs.

	CPP	MW (Da)	Net Charge at pH 7.0	Amino Acid Sequence
Cationic CPPs	Arg8	1267.0	+8	RRRRRRRR
	TAT	1621.9	+8	GRKKRRQRRRPQ
	LMWP	1879.1	+10	VSRRRRRRGGRRRR
Amphipathic CPPs	STR-Arg8	1533.0	+8	Stearyl-RRRRRRRR
	p5RHH	2540.4	+5	VLTTGLPALISWIRRRHRRHC
	RALA	3325.9	+5	WEARLARALARALARHLARALARALRACEA
	Pep-1	2848.2	+3	KETWWETWWTEWSQPKKKRKV
	Penetratin	2248.2	+7	RQIKIWFQNRRMKWKK

**Table 2:** Particle size, polydispersity index and zeta potential of CPP/mRNA complexes.

mRNA/CPP	N/P Ratio	Particle Size (nm)	PDI	Zeta Potential
Arg8	1.5	158.3 (±0.0)	0.09	+16.9 (±0.2)
TAT	3.9	141.7 (±0.9)	0.07	+16.5 (±0.9)
LMWP	1.9	129.5 (±0.3)	0.22	+22.5 (±0.0)
STR-Arg8	7.8	156.8 (±4.7)	0.28	+28.6 (±0.3)
p5RHH	9.7	154.3 (±0.1)	0.20	+16.8 (±0.9)
RALA	9.7	174.6 (±3.7)	0.23	+20.2 (±1.7)
Pep-1	11.6	644.0 (±5.4)	0.40	+7.7 (±0.2)
Penetratin	13.6	487.0 (±5.3)	0.29	+15.7 (±1.0)

## Data Availability

Not applicable.
